# Prognostic significance of baseline T cells, B cells and neutrophil-lymphocyte ratio (NLR) in recurrent ovarian cancer treated with chemotherapy

**DOI:** 10.1186/s13048-020-00661-4

**Published:** 2020-05-15

**Authors:** Jon Røikjær Henriksen, Line Nederby, Frede Donskov, Marianne Waldstrøm, Parvin Adimi, Anders Jakobsen, Karina Dahl Steffensen

**Affiliations:** 1 Department of Oncology, Vejle Hospital - University Hospital of Southern Denmark, Vejle, Denmark; 2 Faculty of Health Sciences, Institute of Regional Health Research, University of Southern Denmark, Odense, Denmark; 3 Biochemistry and Immunology, Vejle Hospital - University Hospital of Southern Denmark, Vejle, Denmark; 4 Department of Oncology, Aarhus University Hospital, Aarhus, Denmark; 5 Department of Pathology, Vejle Hospital - University Hospital of Southern Denmark, Vejle, Denmark

**Keywords:** Ovarian cancer, B cells, T cells, Neutrophils, NLR

## Abstract

**Purpose:**

Biomarkers are needed to guide treatment decisions in recurrent ovarian cancer, as a high proportion of patients do not benefit from treatments. Data on immune subsets in patients receiving chemotherapy are scarce. We investigated the impact of T cells, B cells, neutrophils and the neutrophil-lymphocyte ratio (NLR) in ovarian cancer patients receiving palliative chemotherapy.

**Methods:**

Blood samples were collected prospectively at baseline in recurrent ovarian cancer (*N* = 72) receiving chemotherapy. T cells, B cells, neutrophils, and NLR were analyzed. Primary and secondary endpoints were overall survival (OS) and treatment response, respectively. Cut-offs for T and B cells were predefined.

**Results:**

In patients with low vs. high T and B cells counts, OS was 6.1 months vs 12.0 months (*P* = 0.017) and 6.1 months vs 12.0 months (*P* = 0.011, respectively. Low T and B cells analyzed as continuous variables were also associated with unfavorable OS, *P* = 0.011 and *P* = 0.007, respectively. Neutrophils had no significant prognostic impact. Median NLR was 4.1. High vs. low NLR was associated with poor survival, 7.4 months vs. 15.9 months (*P* = 0.012). In multivariate analysis including platinum sensitivity, number of prior lines of chemotherapy, and performance status, high NLR remained an independent poor prognostic factor HR: 2.17 (95% CI 1.21–3.88) (*P* = 0.009). High NLR was also significantly associated with lack of response, OR 0.15 (95% CI: 0.04–0.51) (*P* = 0.002).

**Conclusion:**

In recurrent ovarian cancer patients undergoing palliative chemotherapy, low T and B lymphocyte counts had an unfavorable prognostic impact. High NLR was associated with lack of response and a poor prognosis, and the parameter may be used in patient counselling and treatment decisions.

## Introduction

Recurrent ovarian cancer represents a therapeutic challenge. Advances in the area of PARP-inhibitors have improved the outcome for some patients [1], but chemotherapy options for platinum-resistant disease or third line treatment are of minimal or no benefit [2, 3]. Checkpoint immunotherapy has been promising in other malignancies, but it has only shown a modest effect as single agent in ovarian cancer [4]. Despite poor efficacy and risk of toxicity from chemotherapy in the recurrent metastatic setting, most patients request further treatment. In these situations, a subjective clinician assessment of a patient’s physical state is the only tool to foresee if a patient will benefit from further chemotherapy. No biomarkers to support the decision exist and are certainly warranted.

The understanding of the immune system in patients receiving chemotherapy is scarce. Still, the notion of tumor infiltrating leukocytes with both tumor promoting and antitumor capabilities places immune cells as potential biomarkers and targets for therapy [5]. In the blood, the neutrophil-lymphocyte ratio (NLR) has been used as a surrogate marker for the balance between an unfavorable impact of neutrophils and a favorable effect of lymphocytes [6]; a high NLR has been associated with poor survival in several malignancies, including ovarian cancer [7–9]. A high NLR value can be obtained by either a high neutrophil count or a low lymphocyte count; a detailed analysis of the relative contribution to the NLR-equation has not been performed in ovarian cancer. Although a few studies have shown a relationship between high baseline neutrophils and poor survival in ovarian cancer [10, 11], the role of circulating neutrophils in the disease is still unclear.

The most abundant type of lymphocyte subset is the T cell. Several studies have shown a positive prognostic impact of a high concentration of tumor-infiltrating T cells in various malignancies, including ovarian cancer [12–21].

Despite their positive prognostic value in the tumor and their contribution to the NLR equation, the impact of circulating T cells as a solitary biomarker has not been investigated in ovarian cancer.

The B cell is another central lymphocyte. They are key elements of the humoral immunity and the source of antibodies. Antibodies against tumor antigens have been found in serum from cancer patients, indicating some B cell antitumor activity [22], while on the other hand, a tumor-promoting function of B cells has been suggested as well [23–27]. The literature on the prognostic impact of tumor-infiltrating B cells has been diverging [28–31], and no investigations have been made on the prognostic impact of circulating B cells in ovarian cancer.

The aim of this study was to quantify major immune subsets included in the NLR equation, neutrophils, T cells, and B cells, in blood from recurrent ovarian cancer patients sampled immediately prior to chemotherapy.

## Methods

### Patient cohort

Study participants were consecutive patients with recurrent metastatic ovarian cancer undergoing palliative chemotherapy at the Department of Oncology, University Hospital of Southern Denmark, Vejle, between December 2016 and October 2018. They were treated according to institutional and national guidelines with the goal of life prolongation and symptom relief. Only non-resectable patients were included. Patients eligible for palliative chemotherapy were included with no further in- or exclusion criteria. Blood samples were drawn 0–7 days prior to commencement of treatment, which was defined as baseline. Imaging was performed every eight to 12 weeks, depending on the treatment regimen, and CA-125 was analyzed routinely at each treatment cycle. The immune cell status had no influence on the choice of treatment.

All patients provided signed informed consent before any study procedure. The Ethics Committee (S-20160049) and the Danish Data Protection Agency (16/28860) approved the study.

### Blood analysis

Enumeration of neutrophils was conducted on a Sysmex XN-9000 instrument (Sysmex, Kobe, Japan) as part of the routine blood testing.

Quantification of lymphocytes, B cells, and T cells was performed by flow cytometry. Fresh venous blood was collected in BD vacutainer EDTA tubes (BD Biosciences, San Jose, CA, USA). Whole blood surface staining was performed in BD Trucount® Tubes using BD Multitest® CD3 FITC/CD16 + CD56 PE/CD45 PerCP/CD19 APC (Clones: SJ25C1, SK7, B73.1, NCAM16.2, 2D1) (BD Biosciences). Subsequently, BD FACS Lysing Solution (BD Biosciences) was used for lysing erythrocytes. Samples were analyzed within 2 h on a BD FACS Canto II (BD Biosciences), and FlowJo version X (Flowjo, Ashland, OR) was used for data analysis.

**Fig. 1:**
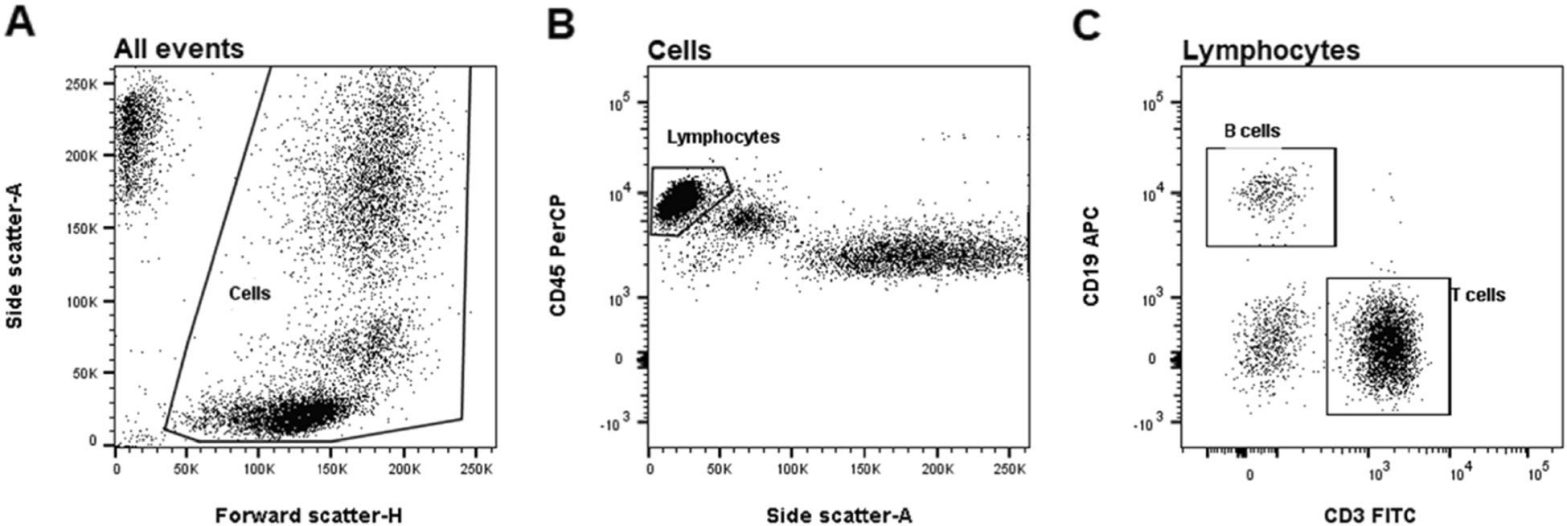
Flow cytometry identifying B and T cells. **a** Forward scatter and side scatter allow for the identification of the cell subset by size and granularity. **b** Side scatter and CD45 staining differentiate the lymphocytes. **c** Fluorochrome-conjugated antibodies enable differentiation of specific cell types in the lymphocyte gate: T cells CD3+ and B cells CD19+

T cells were defined as CD45^high^, side scatter^low^, CD3+, and CD16−/56-. B cells were defined as CD45^high^, side scatter^low^, CD3-, CD16−/56-, and CD19+ (Fig. 1).

Baseline lymphocyte, T, and B cell values were collected in 69 patients. Neutrophil baseline values were obtained in all 72 patients. Missing values were due to either missed blood drawings (*N* = 1) or technical error in sample preparation (*N* = 2).

The applied neutrophil cut-off level originated from a previous report [10], showing baseline neutrophil counts higher than 3.9 × 10^9^ cells/L to be prognostically unfavorable. The T cell cut-off level was based on reference values by Bisset et al. [32], i.e., an abnormally low level of T cells (< 0.536 10^9^ cells/L) and a normal/high level of T cells (≥0.536 10^9^ cells/L). The same reference was used for the B cell cutoff level, i.e., an abnormally low level of B cells (< 0.072 10^9^ cells/L) and a normal/high level of B cells (≥ 0.072 10^9^ cells/L). The cut-off for NLR was defined as the median value.

### Statistical analysis

The Pearson Chi-square test was used to evaluate the association between T cell count, B cell count, neutrophil count, and clinical factors. The primary endpoint was overall survival (OS) calculated from the date of the baseline blood drawing to death or last follow-up.

Kaplan-Meier plots illustrated survival, and the log-rank test analyzed the significance of differences between variables. Median overall survival was used for comparison of survival according to NLR and the level of neutrophil, T cell, and B cell count. Multivariate cox regression analysis tested the independent prognostic significance with the 95% confidence interval (CI). The proportional hazard assumption was tested and complied with all cox regression analyses. Treatment response was defined by either CA-125 or the Response Evaluation Criteria in Solid Tumors (RECIST) 1.1 following the Gynecological Cancer Intergroup (GCIG) criteria [33]. Binary logistic regression tested the relation between treatment response and immune factors. As a secondary aim, immune cell counts were also tested as continuous variables using univariate cox-regression.

Descriptive, correlational, survival, and regression analyses were performed using STATA version 16® (StataCorp, College Station, TX, USA).

## Results

### Patients

The study included 72 patients at a median age of 69 years (range 47–92). High-grade serous carcinoma (HGSC) was the dominating histopathologic type (*N* = 61, 84%). Patients had received one to five lines of chemotherapy prior to inclusion; 32 patients (44%) had received one previous line of therapy. Twenty-four patients (33%) were platinum-sensitive. Table 1 shows the baseline patient characteristics. Baseline CA-125 level, histology, number of previous lines of chemotherapy, present chemotherapy, and performance status were evenly distributed between patients according to immune cell subset level, with the exception that more patients with low baseline B and T cells, respectively, had received more lines of chemotherapy (Table 1). The median OS for all patients was 8.9 months (95% CI: 7.4–15.6). At the time of analysis, 21 patients were still alive with a median follow-up time of 19.3 months (range 11.7–33.3). A total of 21 patients (29%) achieved a treatment response according to the GCIG criteria [33].

**Table 1:** Baseline patient characteristics

	Baseline Cohort (*N* = 72)	High neutrophil count* (*N* = 51)	Low T cell count** (*N* = 19)	Low B cell count*** (*N* = 21)	High NLR**** (*N* = 34)
**Median age**	69	68	71	72	68
(range)	(47–92)	(47–92)	(50–84)	(59–92)	(47–92)
*P value*		*0.852*	*0.248*	*0.116*	*0.657*
**Median CA-125 kU/L**	332	488	330	616	487
(range)	(6–30,072)	(6–30,072)	(11–30,072)	(13–10,325)	(6–30,072)
*P value*		*0.411*	*0.443*	*0.443*	*0.443*
**Histology**					
High-grade serous carcinoma	61 (84%)	45 (88%)	18 (95%)	18 (85%)	31 (88%)
Low-grade serous carcinoma	4 (6%)	3 (6%)	0 (0%)	0 (0%)	1 (3%)
Endometrioid	4 (6%)	2 (4%)	0 (0%)	2 (10%)	2 (6%)
Mucinous	3 (4%)	1 (2%)	1 (5%)	1 (5%)	1 (3%)
*P value*		*0.366*	*0.486*	*0.203*	*0.614*
**Previous lines of chemotherapy**					
1	32 (44%)	22 (43%)	4 (21%)	4 (19%)	11 (32%)
2–3	31 (43%)	23 (45%)	12 (63%)	15 (71%)	19 (54%)
4–5	9 (13%)	6 (12%)	3 (16%)	2 (10%)	5 (14%)
*P value*		*0.391*	*0.068*	*0.011*	*0.122*
**Platinum sensitive**					
No	48 (67%)	33 (65%)	14 (74%)	15 (71%)	25 (71%)
Yes	24 (33%)	18 (35%)	5 (26%)	6 (29%)	10 (29%)
*P value*		*0.582*	*0.446*	*0.579*	*0.395*
**Performance status**					
0–1	46 (64%)	31 (61%)	11 (58%)	14 (66%)	21 (60%)
2	26 (36%)	20 (39%)	8 (42%)	7 (34%)	14 (40%)
*P value*		*0.393*	*0.532*	*0.749*	*0.509*
**Treatment regimen**					
Carboplatin	10 (14%)	9 (18%)	3 (16%)	4 (19%)	8 (23%)
Carboplatin + lipos. Dox.	13 (18%)	8 (16%)	2 (11%)	2 (10%)	2 (6%)
Carboplatin + paclitaxel	1 (1%)	1 (2%)	0 (0%)	0 (0%)	0 (0%)
Liposomal doxorubicin	14 (20%)	10 (19%))	4 (21%)	2 (10%)	8 (23%)
Topotecan	16 (22%)	11 (21%)	6 (31%)	5 (23%)	9 (25%)
Treosulfan	12 (17%)	8 (16%)	2 (11%)	5 (23%)	5 (14%)
Paclitaxel	2 (3%)	2 (4%)	1 (5%)	1 (5%)	1 (3%)
Gemcitabine	2 (3%)	1 (2%)	0 (0%)	0 (0%)	0 (0%)
Vinorelbine	1 (1%)	1 (2%)	1 (5%)	1 (5%)	1 (3%)
Bevacizumab	1 (1%)	0 (0%)	0 (0%)	1 (5%)	1 (3%)
*P value*		*0.642*	*0.579*	*0.251*	*0.062*
**Maintenance treatment#**					
Bevacizumab	8 (11%)	4 (8%)	2 (11%)	1 (5%)	2 (6%)
Olaparib	3 (4%)	3 (6%)	0 (0%)	0 (0%)	0 (0%)
*P value*		0.125	*0.301*	*0.490*	*0.301*

Baseline patient demographics and disease characteristics according to immune cell level. *P* values are derived from chi-square test. * Neutrophil count cut-off 3.9 cells/mL.**T cells count cut-off: 536 cells/μL. *** B cell count cut-off: 72 cells/μL. **** NLR cut-off: 4.1. # Of the 72 patients, 11 recieved maintenance treatment in addition to the primary treatment regimen

### Neutrophils

**Fig. 2:**
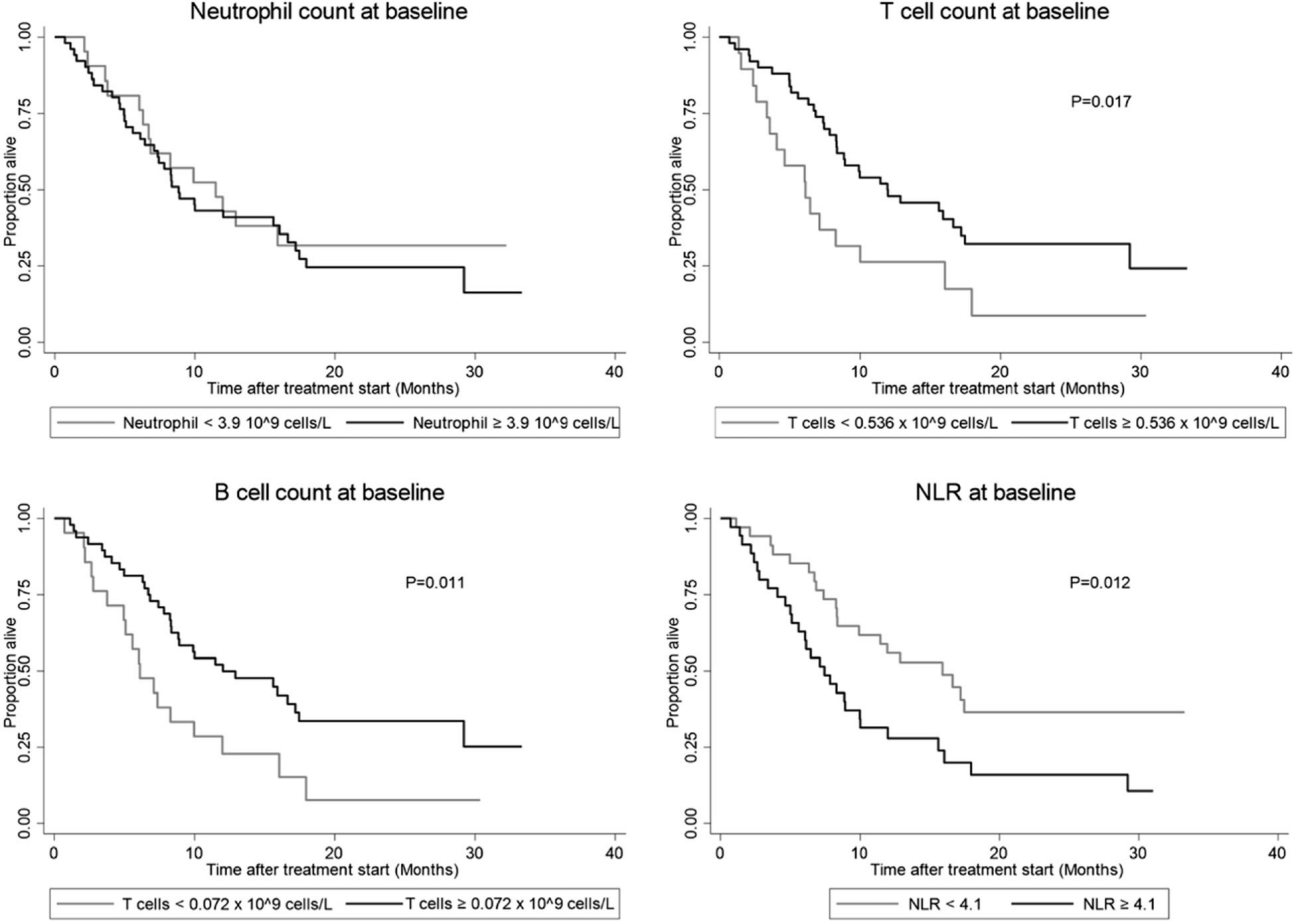
Prognostic impact of baseline immune subsets in recurrent ovarian cancer patients receiving chemotherapy. A favorable prognostic impact was seen with high baseline T cells and B cells. Baseline blood neutrophils had no prognostic impact. High baseline neutrophil lymphocyte ratio (NLR) was associated with a poor prognosis

The median blood neutrophil count was 5.0 × 10^9^ cells/L (range 1.5–20.7). At baseline, 51 patients had a high neutrophil count above the predefined cut-off of 3.9 × 10^9^ cells/L cut-off. The neutrophil count was not significantly associated with OS, neither when analyzed as a dichotomized (Fig. 2) nor as a continuous variable HR: 1.03, (95% CI: 0.96–1.11), (*P* = 0.357).

### T cells

The median blood T cell count at baseline was 0.801 × 10^9^ cells/L (range: 187–2808). A low T cell count (predefined cut-off < 0.536 × 10^9^ cells/L) was associated with poor survival. Patients with low T cell levels (*N* = 19) had median OS of 6.1 months (95% CI: 3.4–10.0) compared to median 12.1 months (95% CI: 8.3–17.2) in patients with normal/high T cell level (*N* = 50) (*P* = 0.017) (Fig. 2). Decreasing T cell count as a continuous variable was significantly associated with poor OS in the Cox regression analysis, HR: 1.09 (*P* = 0.011) (stepwise 100 cells/μL), which corresponds to a 9% increase in risk of death for every decrease of 100 T cells/μL.

### B cells

The median blood B cell count at baseline was 109 × 10^9^ cells/L (range: 12–392). A low B cell count (predefined cut-off < 0.072 × 10^9^ cells/L) was associated with poor survival. Patients with a low B cell level (*N* = 22) had a median OS of 6.1 months (95% CI: 3.8–10.0) compared to 12.0 months in patients with a normal/high B cell level (*N* = 47) (95% CI: 8.3–17.5, *P* = 0.011) (Fig. 2). Decreasing B cell count as a continuous variable was significantly associated with poor OS at baseline HR: 1.05 (*P* = 0.007) (stepwise 10 cells/μL), corresponding to a 5% increase in the risk of death for every decrease of 10 B cells/μL.

### NLR

The median NLR was 4.1 (range 0.9–37.9). High NLR was associated with poor survival. Patients with high NLR (*N* = 35) vs. low NLR (*N* = 34) had a median OS of 7.4 months (95% CI: 5.1–10.0) and 15.9 months (95% CI: 8.3-not reached), respectively, (*P* = 0.012) (Fig. 2). The NLR as a continuous variable was not significantly associated with OS, HR: 1.02 (*P* = 0.121).

### Relation to treatment response

In patients with high NLR, four of 35 (11%) obtained response according to the GCIG criteria, whereas in patients with low NLR, 16 of 34 (47%) achieved response, odds ratio 0.15 (95% CI: 0.04–0.51, *P* = 0.002). No significant association between treatment response and neutrophils, B cells, or T cells was found.

### Multivariate analysis

Multivariate analysis was performed to evaluate the independent significance of the variables. As neutrophils, T cells, and B cells are inherent parts of the NLR, these subsets were therefore not included as single markers in the multivariate analysis. Clinical factors significant in univariate analysis were incorporated (platinum sensitivity, number of prior lines of chemotherapy, and performance status). Histology, CA-125 level, age, and treatment regimen were not significant in univariate analysis and therefore not incorporated. The multivariate analysis revealed high NLR as an independent factor for poor overall survival, HR: 2.17, (95% CI 1.21–3.88), *P* = 0.009, (Table 2).

**Table 2:** Multivariate analysis

	OS	
	HR (95% CI)	*P*
**Platinum sensitive**		
No	*Ref.*	
Yes	0.59 (0.32–1.11)	*0.102*
**Prior lines of chemotherapy**		
1–3	*Ref.*	
4–5	3.82 (1.68–8.72)	*0.001*
**Performance status**		
0–1	*Ref.*	
2	2.93 (1.63–5.28)	*< 0.001*
**NLR**		
Low	*Ref.*	
High	2.17 (1.21–3.88)	*0.009*

Multivariate Cox regression analysis. Hazard ratios (HR) derived from multivariate Cox regression analysis. NLR cut-off defined from the median (4.1)

## Discussion

Recurrent ovarian cancer is marked by an inherent resistance to therapy with low response rates and poor survival. Cancer cell drug-resistance mechanisms play a critical role [34, 35]. A growing notion is that reduced immune surveillance and immune escape also plays a crucial role in cancer progression [36, 37]. Yet, evidence suggests that certain types of chemotherapy may in fact reboost tumor immune response by inducing immunogenic cell death [20]. Evidently, the immune system plays a central role in ovarian cancer, yet immunological investigations have primarily focused on T cells and macrophages infiltrated in the tumor tissue. In this study, we investigated the quantity of essential circulating immune cell subtypes; neutrophils, B cells, and T cells in ovarian cancer with the perspective of improving the treatment strategy.

The present study confirmed the independent prognostic importance of the NLR and also suggested a significant impact of B and T cells. The immune subset analyses revealed that low levels of T cells and B cells were associated with short survival. In contrast, blood neutrophils per se were of no prognostic importance in patients with ovarian cancer. We also demonstrated a high NLR of 4.1 as a strong independent prognostic factor for poor OS and a strong factor for predicting lack of response. Thus, the subset analyses underscored the importance of low levels of T and B cells in the NLR-equation, whereas blood neutrophils did not contribute with significant impact. To the best of our knowledge, a subset analysis has not previously been performed of the contributing factors in the NLR equation in ovarian cancer patients treated with chemotherapy.

Treatment of recurrent ovarian cancer remains a clinical challenge. Most available chemotherapy regimens only have modest effect in terms of response and survival and at the expense of toxicity in most patients. The management of ovarian cancer needs a higher degree of individualization with incorporation of clinically significant factors in the treatment decision making process. This is best exemplified by the use of age as a determining factor. Currently, high age most often affects the choice of treatment in ovarian cancer, although it is not a significant contributing factor in terms of survival, neither in this study nor in previously published literature [38, 39]. In the current study, the significant factors associated with overall survival were number of prior lines of chemotherapy, performance status, and NLR. Performance status is often the only major factor guiding the prediction as to whether the patient will benefit from chemotherapy, but it is subject to poor inter-rater reliability [40]. Hence, the strength of a biomarker such as the NLR compared to performance status is its objective indication. Our data suggest that in addition to performance status, high NLR may be instrumental in patient counseling and involvement in the treatment decision making. Our study showed a high NLR above 4.1 to be associated with a very short survival time. This parameter could be considered in the evaluation of a patient in relation to a new line of chemotherapy.

The focus of the present study was baseline blood values measured just before commencing treatment. The clinical significance of values at this time point is of relevance to the decision of whether to initiate therapy or save patients from ineffective treatment. Usually, these decisions are made after 3 cycles when treatment efficacy is typically evaluated, and patients have undergone 9–12 weeks of treatment already. In clinical practice, it is also relevant to stop ineffective treatment as early as possible, and biomarkers more effective than CA-125 are needed in this setting. Longitudinal sampling of blood could have indicated the potential of the investigated immune cell subsets during treatment, but this was not within the scope of the current study.

Previous studies found an association between a high level of circulating neutrophils and poor survival in cancer patients [10, 11, 41], but the present analysis showed no prognostic impact of the neutrophils count in recurrent ovarian cancer. By contrast, the data were in line with our recent assessment of tumor infiltrating neutrophils in ovarian cancer patients, in which we were unable to identify a negative or positive prognostic impact of the neutrophils [21]. A correlation between high NLR and poor survival in ovarian cancer patients in relation to debulking surgery and adjuvant chemotherapy has been demonstrated [9]. Importantly, in the present study we were able to confirm high NLR as a strong negative prognostic factor in the recurrent setting.

A notion of an anti-tumor effect of lymphocytes is well established in ovarian cancer tissue, while research on the prognostic impact of T cells in the blood has mainly focused on regulatory T cells [42–44] and only sparsely in ovarian cancer [45]. Our results confirmed a significant, favorable prognostic impact of T lymphocyte subsets, both assessed as continuous variables and dichotomized by predefined cut-offs. This encourages more studies on the use of checkpoint inhibitors in ovarian cancer as T cell responses are known to be unleashed by this type of treatment.

The prognostic importance of the B cell count in cancer is unresolved and has mainly been investigated in tumor tissue [28–31, 46]. We used a predefined cut-off and analyzed the prognostic impact both as a continuous and a dichotomized variable. Our analysis pointing towards B cells as contributors to the antitumor activity could indicate an important role of humoral immunity in ovarian cancer, and further research in the area is warranted. An anti-tumor effect of B cells could have implications for future immunotherapy strategies in ovarian cancer since immune checkpoint inhibitors are known to enhance the proliferation of B cells and their production of antibodies [47–50]. Despite disappointing results of single-agent checkpoint inhibitors in ovarian cancer [4, 51], the prognostic importance of B and T cells may support further testing of this group of agents.

Although our study provides interesting results, it has several limitations. Firstly, the small number of patients limits statistical significance and prevents subgroup analysis. In future research, it would be interesting to study the prognostic impact of the investigated immune cells in different histological subgroups, in separate treatment regimens, and in different treatment lines, as the prognostic impact may be conditioned by biological differences in the subgroups. Obviously, chronic illnesses could be hypothesized to affect the immune cell count, and lack of inclusion of this parameter in the current study could be considered a limitation. Also, the types of previous chemotherapy regimens and time elapsed since the last treatment may affect the immune cell count, as patients progressing or recurring during or shortly after previous chemotherapy may be immunologically different. In line with the above mentioned statistical limitations of a relatively small cohort, these factors were not included in order to reduce the number of variables.

Though limited by the patient flow of a small size institution, this study also has strengths. The experiments were conducted prospectively, and analyses were based on predefined cut-offs previously published by others. Furthermore, we were able to confirm the prognostic role of NLR, and our finding that blood neutrophils have no prognostic impact in ovarian cancer is supported by recent intratumoral results from our group [21].

In the context of the limitations of the study, it is essential to emphasize that the current study is exploratory. Full implementation of the results into clinical practice requires verification, preferably in a larger, randomized study.

## Conclusions

In recurrent ovarian cancer patients treated with palliative chemotherapy, low T and B lymphocyte counts had an unfavorable prognostic impact. High NLR was associated with lack of response and a poor prognosis, and the parameter may be used in patient counseling and treatment decisions.

## Data Availability

According to Danish legislation on data protection individual participant data cannot be shared unless fully anonymized. Due to the nature of the stud dataset this is not possible.
